# Renal function-adjusted contrast medium volume is a major risk factor in the occurrence of acute kidney injury after endovascular aneurysm repair: Erratum

**DOI:** 10.1097/MD.0000000000039362

**Published:** 2024-08-09

In the article “Renal function-adjusted contrast medium volume is a major risk factor in the occurrence of acute kidney injury after endovascular aneurysm repair,”^[1]^ which appears in Volume 100, Issue 14 of *Medicine* the correct affiliation a is “College of Medicine, Pusan National University, Division of Vascular and Endovascular Surgery, Department of Surgery, Pusan National University Yangsan Hospital, Yangsan.” Also, the correct corresponding author information is “Dr. Sang Su Lee, MD, PhD, College of Medicine, Pusan National University, Department of Surgery, Pusan National University Yangsan Hospital, 20 Geumo Road, Yangsan, Gyeongsangnam 50612, Republic of Korea (e-mail: phoenixdr@naver.com).”
